# HDACi protects against vascular cognitive impairment from CCH injury via induction of BDNF‐related AMPA receptor activation

**DOI:** 10.1111/jcmm.16770

**Published:** 2021-07-03

**Authors:** Yao‐Ching Fang, Jia‐Yu Hsieh, Amelia Nur Vidyanti, Chih‐Hao Yang, Jing‐Shiun Jan, Kang‐Wei Chang, Chaur‐Jong Hu, Yong‐Kwang Tu

**Affiliations:** ^1^ Taipei Neuroscience Institute Taipei Medical University Taipei Taiwan; ^2^ Department of Neurology Shuang Ho Hospital Taipei Medical University New Taipei City Taiwan; ^3^ Department of Neurology Faculty of Medicine Public Health and Nursing Universitas Gadjah Mada Yogyakarta Indonesia; ^4^ Department of Pharmacology School of Medicine College of Medicine Taipei Medical University Taipei Taiwan; ^5^ Laboratory Animal Center Taipei Medical University Taipei Taiwan

**Keywords:** AMPA, BDNF, CCH, HDAC, OGD, vascular dementia

## Abstract

We previously showed a *hydroxamic acid*‐*based* histone deacetylase i*nhibitor* (HDACi), compound 13, provides neuroprotection against chronic cerebral hypoperfusion (CCH) both in vitro under oxygen‐glucose deprivation (OGD) conditions and in vivo under bilateral common carotid artery occlusion (BCCAO) conditions. Intriguingly, the protective effect of this HDACi is via H3K14 or H4K5 acetylation–mediated differential BDNF isoform activation. BDNF is involved in cell proliferation and differentiation in development, synaptic plasticity and in learning and memory related with receptors or synaptic proteins. B6 mice underwent BCCAO and were randomized into 4 groups; a sham without BCCAO (sham), BCCAO mice injected with DMSO (DMSO), mice injected with HDACi‐compound 13 (compound 13) and mice injected with suberoylanilide hydroxamic acid (SAHA). The cortex and hippocampus of mice were harvested at 3 months after BCCAO, and levels of BDNF, AMPA receptor and dopamine receptors (D1, D2 and D3) were studied using Western blotting analysis or immunohistochemistry. We found that the AMPA receptor plays a key role in the molecular mechanism of this process by modulating HDAC. This protective effect of HDACi may be through BDNF; therefore, activation of this downstream signalling molecule, for example by AMPA receptors, could be a therapeutic target or intervention applied under CCH conditions.

## 1 INTRODUCTION

Vascular dementia (VaD) is the second leading cause of cognitive disorders in older adults after Alzheimer's disease (AD). The causes of VaD are multifactorial with complex pathophysiologies. For that reason, effective treatments for VaD in humans are lacking. Hence, establishment of the model of chronic cerebral hypoperfusion (CCH), which is a major cause of VaD, was used to evaluate effectiveness of pharmacological intervention.

We previously showed that *hydroxamic acid*‐*based* histone deacetylase i*nhibitors* (HDACi) provides neuroprotection in vivo under CCH conditions.^1^ CCH is associated with cognitive impairment linked to vascular disease^2^ and is mainly due to inflammation and subsequent neuronal apoptosis.^3^, ^4^ The protective effect of these HDAC inhibitors was through H3K14 or H4K5 acetylation–mediated differential brain‐derived neurotrophic factor (BDNF) isoform activation has been shown in vitro,^5^ leading to increase BDNF concentration and improve cognitive function. However, the HDACi‐induced BDNF activation and the downstream molecular mechanism affecting cognitive function remains to be clarified.

In vitro studies have shown that the activation of BDNF‐TrkB signalling increases the synaptic delivery of Ca^2+^‐permeable AMPA receptors (CP‐AMPARs).^6^, ^7^ Modulation of AMPARs trafficking plays a key role in synaptic plasticity, learning and memory.^8^, ^9^, ^10^ Furthermore, an acute injection of exogenous BDNF into the nucleus accumbens (NAc) core of adult rats rapidly (30 minutes) up‐regulates the GluA1‐subunit of AMPAR surface expression in the NAc core through protein synthesis and an ERK‐dependent mechanism.^11^, ^12^

Moreover, prior studies demonstrated that BDNF regulates the expression of dopamine (D1 and D3) receptors.^13^, ^14^ In addition, BDNF along with dopamine D2 receptor have interplay and involved in the pathophysiology of various neuropsychiatric diseases.^15^ The activation of those dopamine receptors is important for memory consolidation and plays a role in hippocampal plasticity.^16^

Therefore, this study aimed to examine whether BDNF activation by HDACi may affect the protein levels of AMPA and dopamine receptors under CCH conditions, thus leads to attenuation of cognitive impairment.

## 2 MATERIALS AND METHODS

### 2.1 Animals

Male C57BL/6J mice (16 weeks old, weighing 25‐35 g, Bio‐Lasco Taiwan Co., Ltd) were used in all experiments. The temperature (22 ± 1°C) and humidity (55% ± 10%) in the environment of mice were controlled, and they were subjected to a 12‐hour light/dark cycle (lights on at 07:00). Food and water were given ad libitum to all mice throughout the experiments. Animal care and experimental procedures in this study were conducted in accordance with the Guidelines for the Care and Use of Laboratory Animals from the Ethics Committee of Taipei Medical University.

### 2.2 Bilateral common carotid artery occlusion surgery

To induce CCH injury, bilateral common carotid artery occlusion (BCCAO) surgery was conducted following a previous procedure.^17^ Briefly, the parietal skulls of C57BL/6J mice were exposed to measure the baseline cerebral blow flow (CBF) using laser Doppler flowmetry. Bilateral CCAs were then carefully separated, and the right CCA was occluded for 1 week. The procedure was then repeated for the left CCA: Steps used were similar, except for transient ligation of the left CCA for 30 minutes. The CBF was measured to ensure that it had reduced by 80%–90% from baseline. The sham surgery procedure was similar to that of BCCAO, but neither CCA was ligated. DMSO, compound 13 or SAHA were given intraperitoneally (25 mg/mL) once every 2 days for 3 months, beginning 2 days after CCA ligation. The mice were grouped as sham control (n = 4), BCCAO injected with DMSO (n = 4), BCCAO injected with compound 13 (n = 4) and BCCAO injected with SAHA (n = 4).

### 2.3 Western blot analysis

Proteins were resolved based on molecular weight through electrophoresis on 8%, 10% and 12% polyacrylamide gels, followed by transfer to a polyvinylidene difluoride membrane. The membrane was blocked and incubated with antibodies for neurotrophic factor (BDNF), α‐amino‐3‐hydroxy‐5‐methyl‐4‐isoxazolepropionic acid receptor (AMPAR), dopamine receptor (D1, D2 and D3) and beta‐actin. The protein levels were analysed using an enhanced chemiluminescence detection kit (GE Healthcare).

### 2.4 Immunohistochemistry

Three months after BCCAO and sham surgery, respectively, the mice were anesthetized and intracardially perfused with phosphate‐buffered saline (PBS), followed by 4% paraformaldehyde. Their brains were removed, postfixed in 4% paraformaldehyde at 4°C and then stored in 30% sucrose in 0.1 mol/L PBS (pH 7.4). Serial coronal sections were cut using a cryostat, and coronal sections were treated with 5% normal donkey serum and then incubated in a primary antibody solution overnight at 4°C. After being washed, the samples were incubated in a secondary antibody solution containing donkey anti‐rabbit or donkey anti‐goat conjugated with fluorescein (1:200, Zhongshan Biotechnology) for 1 hour at room temperature. All images were acquired using fluorescence microscopy (Olympus IX70) or light microscopy (Olympus).

## 3 RESULTS

### 3.1 HDACi rescued the reduction in BDNF‐positive cells within the hippocampus of CCH mice

**FIGURE 1:**
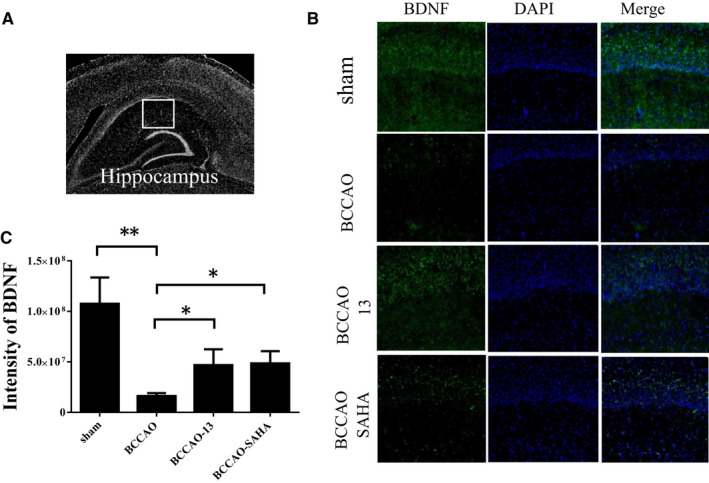
Increased BDNF in the hippocampus at 3 mo following chronic cerebral hypoperfusion (CCH). A and B, The region and immunostaining of BDNF in the hippocampus. C, Semiquantitative analytic data of immunostaining image. Each bar represents the mean ± SEM of four independent experiments (n = 4, *P <.05, **P <.01 vs indicated group)

Previous studies have shown the protective effects of HDACi on vascular cognitive impairment induced by CCH via histone acetylation^1^ and in OGD conditions via differential BDNF isoform expression.^5^ We subsequently investigated which spatial regions of the protein levels of BDNF would be affected by HDACi under CCH injury. Although Western blotting analysis has shown that SAHA increases the protein levels of BDNF under ischemic conditions,^18^ to our knowledge, the levels of it were never examined under CCH condition with immunohistochemistry analysis. The nuclei were labelled with DAPI, and the BDNF‐expressing cells were measured 3 months after treatment in hippocampus (Figure 1A). The results showed that the BDNF‐positive cells were significantly decreased in CCH mice administrated with DMSO compared with those without BCCAO (Figure 1B). For CCH mice injected with compound 13 or SAHA at 25 mg/kg, there was an increase in BDNF‐positive cells compared with the DMSO group. The potency of BDNF induction with our HDACi in CCH mice has a similar effect to that of an already marketed drug, SAHA (Figure 1C). Therefore, this synthetic HDACi compound can be considered to be a putative *BDNF* inducer, and its protective effects might be used in other neurodegenerative disorders.

### 3.2 HDACi had no effect on BDNF‐positive cells within the cortex of CCH mice

**FIGURE 2:**
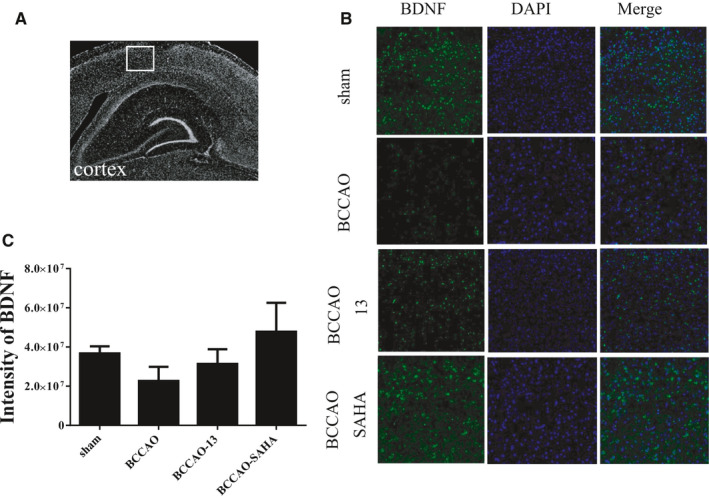
Effect of compound 13 on the levels of BDNF in the cortex of CCH mice. A and B, The region and immunostaining of BDNF in the cortex. C, Semiquantitative analytic data of immunostaining image. Each bar represents the mean ± SEM of four independent experiments (n = 4, *P <.05, **P <.01 vs indicated group)

Immunohistochemical analysis was conducted to examine whether HDACi induced BDNF activation in the cortex under CCH conditions, and the protein level of BDNF in the cortex was thus investigated (Figure 2A). SAHA, the FDA‐approved drug, was also used as the positive control, as it has a protective effect in ischaemia conditions via the induction of BDNF.^18^ In the cortex of CCH mice, the protein level of BDNF was slightly, but not significantly decreased compared with that of DMSO, and compound 13 showed no BDNF changes (Figure 2B and C). This implies that this compound, which belongs to hydroxamic acid–derived HDACi, has an effect on the protein levels of BDNF *within the hippocampus but not within the cortex*.

### 3.3 HDACi increased AMPAR (GluA1) expression but not dopamine receptors in the hippocampus of CCH mice

**FIGURE 3:**
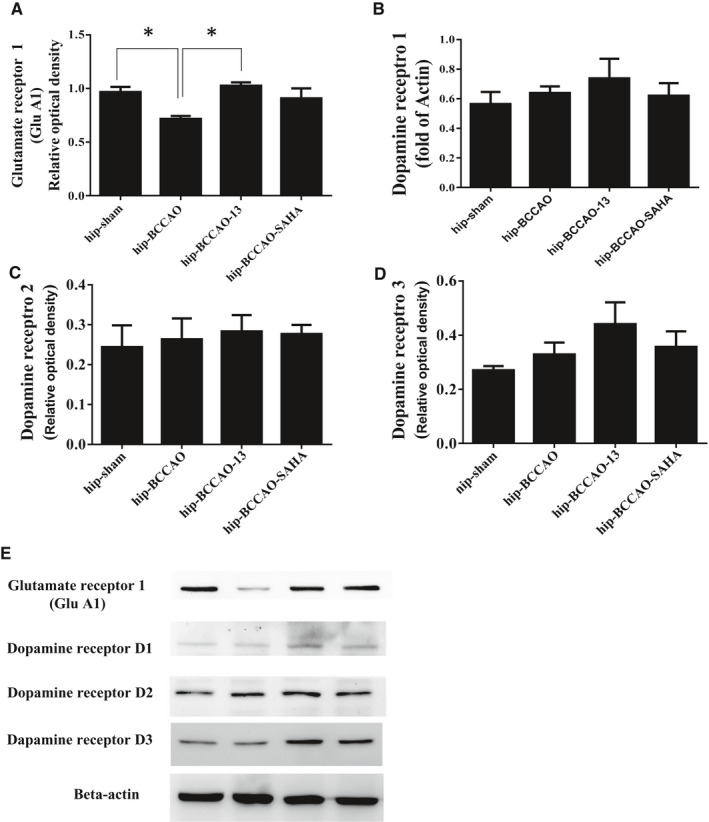
Effect of compound 13 on the levels of glutamatergic, and dopaminergic receptor in the hippocampus (H) of CCH mice. The protein levels of AMPA receptor A, and dopamine receptors 1 B, 2 C and 3 D were measured statistically after BCCAO for 3 mo. β‐actin served as the loading control. A representative Western blot showing the levels of AMPA receptor, and dopamine receptors in the hippocampus E. The densities were normalized to β‐actin, and each bar represents the mean ± SEM of independent experiments (*P < .05, **P < .01 vs the indicated group)

We next attempted to clarify the molecular mechanisms associated with BCCAO‐induced CCH following HDACi administration. BDNF activation following HDACi administration is believed to transform epigenetic information into a stable long‐term memory. Changes in gene expression following HDACi administration are essential mechanisms involved in modulating synaptic plasticity. We evaluated the AMPAR or dopamine receptor expression in the hippocampus of CCH mice following HDACi injection (Figure 3A). For mice that received bilateral BCCAO occlusion, AMPAR expression was significantly decreased in the DMSO group compared with the sham group, whereas the protein levels of the AMPA receptor (GluA1) were significantly increased in CCH mice that received the HDACi injection (i.p.) compared with the DMAO control. SAHA has a similar effect with respect to increasing GluA1 (Figure 3B and C). We also detected dopamine receptors (D1R, D2R or D3R) in the compound 13–injected mice but found no changes in the protein levels of these three types of dopamine receptor in these mice compared with the DMSO‐injected ones. No changes were detected in the SAHA mice compared with the DMSO group.

### 3.4 HDACi injection did not alter the protein level of GluA1 and dopamine receptor 1,2 or 3

**FIGURE 4:**
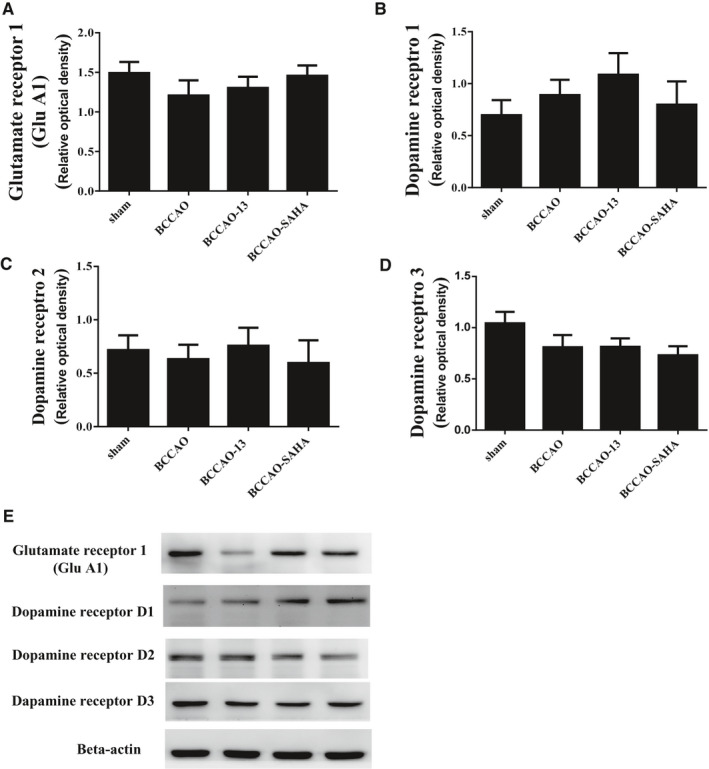
Effect of compound 13 on the levels of glutamatergic, and dopaminergic receptor in the cortex of CCH mice. The protein levels of AMPA receptor A, and dopamine receptors 1 B, 2 C and 3 D were measured statistically after BCCAO for 3 mo. β‐actin served as the loading control. E, Representative Western blot images of AMPA receptor and dopamine receptors in the cortex. The densities were normalized to β‐actin, and each bar represents the mean ± SEM of independent experiments (n = 4, *P < .05, **P < .01 vs the indicated group)

We also clarify the molecular mechanisms associated with BCCAO‐induced CCH following HDACi administration in the cortex of CCH mice (Figure 4A). To better understand the BDNF response after HDACi injection, we used Western blotting analysis to identify the protein levels of the AMPARs (GluA1) and differential dopamine receptors (D1‐D3) in mice given this injection compared with the DMSO‐injected CCH mice. HDACi‐relevant spatial brain regions were chosen based on our previous findings, which showed rescued cognitive impairment during CCH. The rescue of cognitive impairment could be associated with an increased transcriptional response involving differential regulated genes related to the glutamate receptor, dopamine receptors or synaptic proteins. We thus examined the encoding proteins, including those of the glutamate receptor (GluA1) and dopamine receptors (D1R, D2R or D3R). The data showed glutamate receptor (GluA1) or dopamine receptors in the cortex of mice with compound 13 or SAHA injection had no changes compared with the DMSO‐injected ones (Figure 4B and C).

### 3.5 Proposed mechanism by which HDACi protects from injuries via BDNF‐AMPAR signalling

One HDACi in particular, compound 13, increases the acetylation status in histones 3 and 4, which further react with specific promotors of *BDNF*.^5^ BDNF acted as a modulator to induce changes in gene expression or protein synthesis. The vesicle‐released BDNF binds to the TrkB receptors and alters protein levels of AMPA receptors, but not dopamine receptors. This might be via the regulation of transcriptional or translational machinery to up‐regulated protein synthesis.

**FIGURE 5:**
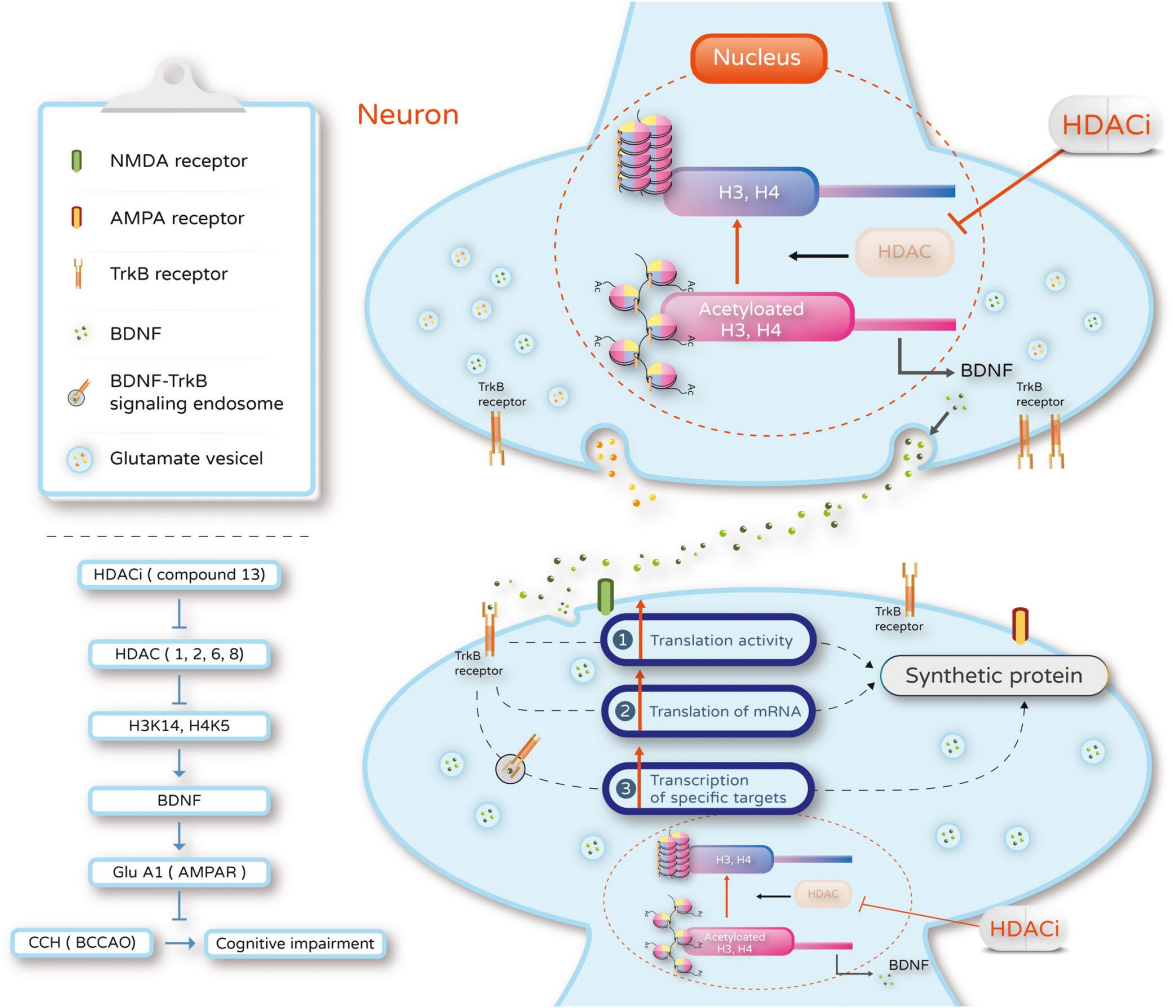
Proposed mechanism by which the HDACi‐induced brain‐derived neurotrophic factor and AMPA receptor activation mediate cognitive function in CCH mice

HDACi might protect against VCI from CCH injury via induction of BDNF‐related AMPAR activation (Figure 5).

## 4 DISCUSSION

Our previous studies have shown that HDACi protects against CCH‐induced hippocampal atrophy, prevents cognitive dysfunction and affects the acetylation status of H3K14 and H4K5, thereby increasing the expression of different BDNF isoforms. Therefore, HDACi may be an effective treatment for reducing CCH‐related cerebrovascular diseases.^1^ However, the signalling process following BDNF induction by HDACi in relation to affecting cognitive function requires further research. In this study, we used a CCH mouse model related to vascular cognitive impairment, and the results showed that CCH significantly reduced the expression of BDNF‐positive cells (Figure 1) and followed by the decrease levels of AMPARs in the hippocampus (Figure 3). Furthermore, HDACi significantly increases in the expression of BDNF and AMPARs as seen in BCCAO mice. The compound 13 is more effective than SAHA in regulating AMPAR expression (Figure 3). These results indicate that HDACi is essential for histone acetylation. HDACi induced an increase in levels of BDNF and AMPARs, which protect against hippocampal atrophy and cognitive impairment following CCH. To our knowledge, this is the first study to demonstrate that HDACi may increase AMPARs under CCH conditions.

We realize that HDACi induces BDNF induction, and its downstream signalling involves multiple pathways relating to various receptors (eg glutamate and dopamine receptors). In the biosynthesis of BDNF, the pre‐pro‐BDNF is synthesized and folded in the endoplasm^19^ and is then transferred to the Golgi apparatus and to be cut into pro‐BDNF. Pro‐BDNF is further cleaved by differential protease to form mature BDNF (m‐BDNF). Pro‐BDNF may be considered an important regulator of brain function during development,^20^ and m‐BDNF has been shown to participate in protection and synaptic plasticity during adulthood.^21^

Mature BDNF can execute its functions through PI3K‐AKT, MAPK(ERK), PLC‐r or GTPase. We did not specifically explore these paths in our study; instead, we explored the receptors that may be affected by these paths downstream.

The differential BDNF isoforms that affect the cognition function through specific signal transduction require investigation. Through these signal cascades, BDNF can regulate the development and survival of neurons, mediate various dendrite and dendritic spines reorganization processes and regulate synaptic function and plasticity, such as LTP related to learning and memory. Experimental evidence has also shown that deleting the TrkB or BDNF gene leads to atrophy of dorsal forebrain cells, dendritic mutation and neuronal loss.

There is convincing evidence that CCH is involved in VaD‐related neuropathological and cognitive impairment characteristics.^3^ HDACi can mitigate the progression of neurodegenerative diseases by inhibiting oxidative stress and neuroinflammation. HDACi has also been shown to reduce Aβ deposition and block GSK‐3β in AD animal models, thereby promoting the recovery of cognitive function.^22^, ^23^ In our previous study, the administration of HDACi during CCH almost completely reversed these effects.^1^ Moreover, HDACi has been shown to stimulate cell proliferation and the production of new neurons in the adult hippocampus,^24^ which may help reduce the observed hippocampal atrophy of CCH‐injected mice. HDACi also has antioxidant potencies in focal cerebral ischaemia,^25^, ^26^ and it can inhibit neuroinflammation and glial cell activation. The mechanisms underlying the protective effects of HDACi in reducing ischemic neuronal damage require further study. BDNF plays a role in neurotransmission, the establishment of neural circuits and neuroplasticity and is thus essential for learning and memory.^27^ Insufficient BDNF release has been confirmed in a variety of neurological diseases, such as traumatic brain injury, stroke and AD.^28^, ^29^, ^30^ In this study, the protein levels of BDNF were significantly reduced in CCH mice compared with the sham group, and this result is consistent with those of previous studies.^1^ Evidence has also shown that stellate cells may release BDNF to support neuronal survival and learning and learning‐related plasticity.^31^

Followed by BDNF activation, glutamate receptors could be induced. One type of glutamate receptor, AMPAR, is composed of four types of subunits coded by different genes, namely *GRuA1*, *GluA2*, *GluA3* and *GluA4*, which combine to form a tetramer. Two types of subunits in the assembly of AMPAR tetramers could be hetero‐tetramers by two differential dimers (GluA1‐GluA2 or GluA2‐GluA3) and homo‐tetramer receptors (GluA1‐GluA1) by the same two dimers. The heterotetrameric receptors have a linear‐voltage (IV) relationship and are impermeable in Ca^2+^, and homotetramer receptors show high conductance and rapid decay kinetics and are permeable in Ca^2+^. Thus, homotetramer receptors are usually referred to as Ca^2+^‐permeable AMPARs (CP‐AMPARs).^32^ CP‐AMPARs, the majority of which are likely to be GluA1 homodimers, are broadly detected in synapses.

Many researchers have reported the appearance of CP‐AMPARs at specific synapses during the induction of certain forms of synaptic plasticity, including long‐term potentiation (LTP) associates with memory consolidation, or following certain behavioural manipulations, such as fear conditioning.^33^, ^34^ These receptors have high conductance and enhance synaptic transmission, and they may activate unique Ca^2+^‐sensitive signalling pathways related to LTP.^35^, ^36^, ^37^ The main difference between each of the AMPAR subunits is their C‐terminal sequences, and these determine their interaction with the differential scaffold protein. All AMPARs contain PDZ domains, which interact with different synaptic proteins. For example, PDZ of GluA1 binds to SAP97, and GluA2 conjugates to GRIP or PICK1. PDZ domain binds to the synaptic protein, PSD‐95, through stargazin.^38^, ^39^ A growing body of evidence also shows that BDNF can induce multiple pathways and that AMPARs are the possible substrates of multiple signalling molecules and may be related to LTP.

In this study, compound 13 is superior to SAHA in regulating AMPAR expression. compound 13 is a (*E*)‐3‐(4‐(((3‐(3‐chloro‐10,11‐dihydro‐5*H*‐dibenzo [b,f]azepin‐5‐yl)propyl)amino)methyl)phenyl)‐N‐hydroxy‐acrylamide, behaving as histone deacetylase inhibitor. It is synthesized using SAHA as the base structure.^1^ SAHA exerts protective effect by inducing anti‐apoptosis and anti‐inflammatory properties on ageing animals. It decreases the activation of cleaved caspase‐3, inducible nitric oxide synthase (iNOS) and N‐methyl‐D‐aspartate (NMDA) receptor‐calcium/calmodulin dependent kinase II (CaMKII) pathway.^40^ Meanwhile, in the present study we used CCH model correlated with vascular cognitive impairment. In this model, the impairment of cognitive function was associated with a reduction in cerebral blood flow and hippocampal atrophy.^17^ In addition to increase the expression of AMPAR, compound 13 has shown its effectiveness to restore cerebral blood flow as well as improves hippocampal atrophy.^1^

In conclusion, our study proposes that modulation of AMPAR by HDACi may provide benefit as therapeutic intervention in patients with vascular cognitive impairment. Nevertheless, this study has some limitations. First, the administration of compound 13 as HDACi was only given for 3 months. Observation for longer period is needed to evaluate the long‐term efficacy and adverse reaction. Second, the animal model used in our study was categorized as mature adult mice, which corresponds with human during their thirties. Meanwhile, vascular cognitive impairment or VaD is a condition faced during late life or middle age in human, which best corresponds with mice age at least 14 months old. Therefore, any discrepancy related to ageing process is possible and may result different findings. Future studies need to investigate the long‐term effect of HDACi in the old animal model of VaD.

## CONFLICT OF INTEREST

The authors declare no potential conflicts of interest.

## AUTHOR CONTRIBUTIONS

**Yao‐Ching Fang:** Writing‐original draft (lead); Writing‐review & editing (equal). **Jia‐Yu Hsieh:** Methodology (lead). **Amelia Nur Vidyanti:** Methodology (equal). **Chih‐Hao Yang:** Methodology (equal). **Jing‐Shiun Jan:** Methodology (equal). **Kang‐Wei Chang:** Methodology (equal). **Chaur‐Jong Hu:** Writing‐review & editing (equal). **Yong‐Kwang Tu:** Writing‐review & editing (equal).

## Data Availability

The data that support the findings of this study are openly available in figshare at https://figshare.com/s/6dfc8548774680184346.
